# SafeRx: A Canonical Pharmaceutical Knowledge Integration Framework for Multimodal Medication Safety

**DOI:** 10.3390/bioengineering13070763

**Published:** 2026-06-30

**Authors:** Andrei-Flavius Radu, Ada Radu, Bogdan Uivaraseanu, Delia Mirela Tit, Cosmin Mihai Vesa, Gabriela S. Bungau

**Affiliations:** 1 Doctoral School of Biological and Biomedical Sciences, University of Oradea, 410087 Oradea, Romania; andreiflavius.radu@uoradea.ro (A.-F.R.); dtit@uoradea.ro (D.M.T.); cosmin.vesa@csud.uoradea.ro (C.M.V.); gbungau@uoradea.ro (G.S.B.); 2 Department of Psycho-Neuroscience and Recovery, Faculty of Medicine and Pharmacy, University of Oradea, 410073 Oradea, Romania; 3 Department of Pharmacy, Faculty of Medicine and Pharmacy, University of Oradea, 410028 Oradea, Romania; 4 Department of Surgical Disciplines, Faculty of Medicine and Pharmacy, University of Oradea, 410073 Oradea, Romania; 5 Department of Preclinical Disciplines, Faculty of Medicine and Pharmacy, University of Oradea, 410073 Oradea, Romania

**Keywords:** medication safety, drug–drug interactions, clinical decision support, semantic harmonization, pharmacovigilance, knowledge integration, interoperability, provenance-aware systems

## Abstract

Medication-related harm remains a major patient-safety challenge, substantially driven by fragmented pharmacovigilance ecosystems, inconsistent drug nomenclature, heterogeneous interaction knowledge bases, and the absence of unified multimodal medication-safety infrastructures. Existing clinical decision-support systems frequently remain interaction-centered, with limited integration of complementary safety domains such as lactation risk assessment, intravenous compatibility evaluation, and regulatory toxicity overlays. This study aimed to develop and evaluate SafeRx, a provenance-aware pharmaceutical knowledge integration platform designed to harmonize heterogeneous medication-safety data within a unified canonical framework. SafeRx integrated Romanian regulatory product summaries, Danish drug–drug interaction repositories, OpenFDA boxed warning datasets, LactMed lactation safety records, and Stabilis intravenous compatibility data through automated extraction pipelines, ontology-aware canonical substance normalization, AI-assisted semantic enrichment, and pharmacist-supervised governance workflows. The platform consolidated 2190 canonical substances linked to 9463 validated identifier mappings, 9932 canonical interaction pairs enriched with tier-based prioritization and mechanism-aware annotations, 514 regulatory boxed warning records, 1895 lactation safety entries, and 9088 intravenous compatibility records. SafeRx demonstrates the feasibility of constructing an interoperable, explainable, and pharmacist-supervised medication-safety infrastructure capable of integrating heterogeneous regulatory, pharmacologic, reproductive, and physicochemical safety domains within a unified clinically navigable framework for research, pharmacovigilance, and clinical decision-support applications. Future studies should evaluate prospective clinical implementation, electronic health record interoperability, and the real-world impact of tier-based prioritization on prescribing workflows, alert fatigue, and medication-safety outcomes.

## 1. Introduction

Medication-related harm remains a major and largely preventable patient-safety challenge, particularly in polypharmacy, high-risk prescribing, intensive care, and transitions of care. Medication errors frequently arise from fragmented workflows, heterogeneous information systems, and incomplete integration of clinically relevant safety data across prescribing and administration processes. As therapeutic complexity continues to increase, medication-safety infrastructures increasingly require interoperable and clinically interpretable decision-support environments capable of consolidating heterogeneous pharmaceutical safety information within unified clinical workflows [1,2].

The growing prevalence of multimorbidity and polypharmacy has substantially increased exposure to adverse drug events, particularly among vulnerable populations requiring multiple concurrent medications. Complex medication regimens are associated with elevated risks of drug–drug interactions (DDIs), adverse drug reactions, and hospitalization, creating clinically challenging prescribing scenarios that demand context-aware decision support [3]. Clinical decision-support systems (CDS) have been consistently associated with reductions in medication errors when integrated within prescribing workflows; however, CDS effectiveness remains strongly influenced by workflow integration, contextual relevance, usability, and broader sociotechnical factors [4].

Despite these advances, medication-safety assessment is increasingly challenged by fragmented pharmacovigilance ecosystems in which clinically relevant drug-safety information remains distributed across heterogeneous regulatory, clinical, and pharmacologic data sources. Persistent limitations related to inconsistent drug nomenclature, fragmented provenance, variable severity interpretation, and limited multimodal integration have been documented across existing DDI infrastructures [5].

Cross-platform assessments of major commercial DDI knowledge bases have demonstrated substantial variability in interaction coverage, severity classification, alert burden, and editorial inclusion policies, with inconsistent standardization contributing to alert variability, interoperability limitations, and reduced clinical relevance [6]. Consensus recommendations have further emphasized that heterogeneous evidence-evaluation strategies and variable editorial policies may generate excessive alert burden and poor reproducibility across medication-safety infrastructures, reinforcing the need for transparent, systematically governed, and clinically actionable DDI frameworks [7,8].

Comparative evaluations of widely used DDI checkers have demonstrated substantial discrepancies in interaction classification across platforms such as Drugs.com, Medscape, DrugBank, and DDInter, together with limited coverage of newly approved antimicrobials, highlighting the need for more standardized and continuously updated medication-safety infrastructures [9].

Semantic interoperability limitations compound these challenges further. Structural inconsistencies between major biomedical drug terminologies, including RxNorm and SNOMED CT, substantially limit cross-system knowledge exchange and accurate integration of medication-safety information across heterogeneous clinical infrastructures [10]. Evaluations of common data models, including OMOP, Sentinel, and PCORnet, have similarly demonstrated persistent challenges related to heterogeneous terminology coverage, lossy semantic transformations, and inconsistent preservation of clinically relevant associations during medication-data harmonization workflows [11]. Free-text drug nomenclature, multilingual terminology variation, and inconsistent ingredient mapping further impair reproducibility and transparency across pharmacovigilance datasets, highlighting the importance of canonical, provenance-aware drug-mapping frameworks [12,13].

Alert fatigue represents a well-documented consequence of these limitations. Usability-focused evaluations have demonstrated that excessive interruptive alerting, limited patient-specific contextualization, and poor prioritization strategies substantially contribute to clinician dissatisfaction and high override rates [14]. ICU-focused CDS studies have further shown that tailoring alerts according to clinical relevance and care-setting specificity may significantly reduce high-risk drug combinations and mitigate alert fatigue, reinforcing the importance of context-aware and clinically prioritized medication-safety infrastructures [15,16]. Pharmacists frequently rely on multiple fragmented drug-information resources with inconsistent coverage and limited interoperability, while pharmacist-integrated medication review has been shown to substantially reduce prescribing errors in high-risk clinical environments [17,18].

Beyond conventional DDI assessment, several complementary medication-safety domains remain poorly integrated within existing clinical decision-support environments. Intravenous physicochemical incompatibilities represent frequent yet under-recognized safety events in intensive care and hematology settings, where polymedicated patients require multiple concurrent infusions through limited vascular access. Fragmented compatibility evidence, concentration-dependent administration risks, and the absence of integrated compatibility-support infrastructures complicate real-time bedside assessment and may contribute to clinically significant morbidity, including pulmonary toxicity, systemic inflammatory responses, and microvascular impairment [19,20]. Medication safety during breastfeeding similarly remains underserved, with large proportions of medications lacking robust lactation-risk and infant-exposure data. Comparative analyses of FAERS and LactMed^®^ have demonstrated important differences between exploratory pharmacovigilance signals and clinically curated breastfeeding recommendations, highlighting the need for integrated lactation-safety infrastructures within clinical decision-support environments [21,22].

Recent advances in AI-driven CDS systems, knowledge graphs, and pharmacovigilance analytics have substantially expanded DDI detection capabilities. Nevertheless, current infrastructures remain constrained by heterogeneous severity frameworks, limited interoperability, insufficient explainability, and incomplete integration of vulnerable-population safety domains [23,24]. Benchmarking studies have further demonstrated that LLM-based medication-safety systems may struggle to reliably identify context-dependent contraindications and clinically relevant DDIs, emphasizing the importance of explainable, provenance-aware, and human-supervised medication-safety architectures [25]. A bibliometric analysis of 19,151 DDI publications further confirmed the rapid expansion of DDI research while simultaneously revealing persistent fragmentation across pharmacologic, clinical, and computational research domains. These findings reinforce the need for unified and provenance-aware medication-safety infrastructures integrating heterogeneous evidence sources within clinically navigable frameworks [26]. Existing platforms frequently remain interaction-centered, with limited incorporation of complementary safety domains such as lactation risk assessment, intravenous compatibility evaluation, and regulatory toxicity overlays within unified decision-support environments [6,27].

To address these limitations, our team engineered SafeRx, a provenance-aware pharmaceutical knowledge integration and clinical decision-support platform designed to harmonize heterogeneous medication-safety information from regulatory, pharmacologic, and clinically curated sources within a unified canonical framework. Unlike conventional product-centered or editorially opaque interaction systems, SafeRx was implemented as a substance-centric infrastructure integrating canonical pharmaceutical identity resolution, cross-source interaction harmonization, AI-assisted semantic enrichment, regulatory boxed warnings, LactMed-derived lactation safety information, and intravenous compatibility data within a single clinically interpretable environment. Automated extraction pipelines, ontology-aware normalization strategies, and conflict-aware interaction consolidation were combined while preserving explicit source attribution and enrichment provenance throughout the processing pipeline. A pharmacist-supervised validation and governance architecture was additionally incorporated to support expert review, human-in-the-loop clinical oversight, and longitudinal quality assurance of generated interaction annotations.

Our research focused in developing and evaluating SafeRx as a scalable, transparent, and multimodal medication-safety infrastructure capable of supporting interoperable pharmaceutical knowledge integration, clinically actionable interaction prioritization, and provenance-preserving decision support across heterogeneous biomedical datasets.

## 2. Materials and Methods

### 2.1. System Architecture and Platform Design

**Figure 1:**
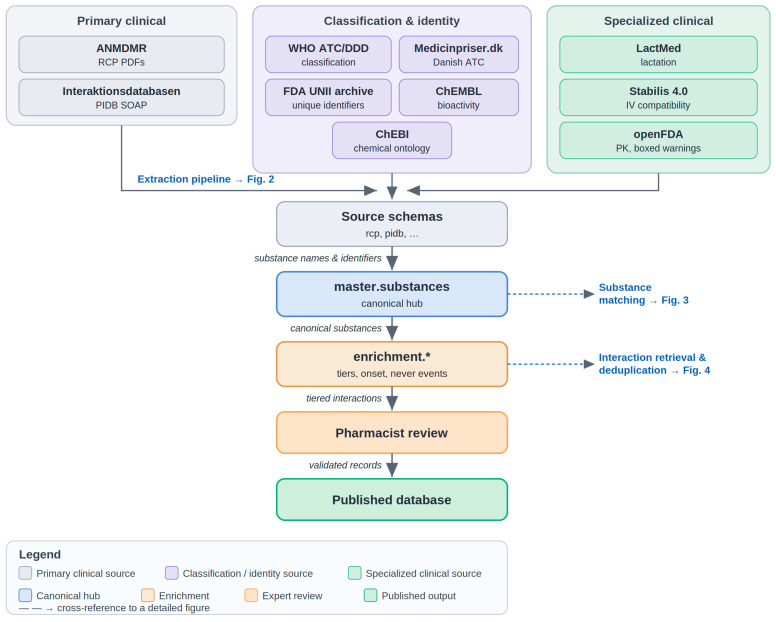
Architectural framework and data-processing pipeline of the SafeRx platform. The diagram illustrates the ingestion of heterogeneous data sources, the substance-centric identity resolution, and the integration of AI-assisted enrichment with pharmacist-led governance. ANMDMR, Romanian National Agency for Medicines and Medical Devices; RCP, Summary of Product Characteristics; PIDB, Interaktionsdatabasen (Danish Drug Interaction Database); ATC/DDD, Anatomical Therapeutic Chemical/Defined Daily Dose classification system; UNII, Unique Ingredient Identifier; ChEBI, Chemical Entities of Biological Interest; PK, pharmacokinetic.

SafeRx was developed as a unified pharmaceutical knowledge integration and clinical decision-support platform designed to aggregate, normalize, enrich, and validate heterogeneous medication-related safety data originating from multiple regulatory, pharmacologic, and clinical evidence sources. The overall system architecture, integration workflow, and major processing layers implemented within the platform are summarized in Figure 1.

The platform architecture integrated automated extraction pipelines, canonical substance normalization, cross-source identity resolution, semantic enrichment workflows, provenance-aware aggregation strategies, and pharmacist-supervised validation mechanisms within a unified interoperable pharmaceutical knowledge framework.

Unlike conventional interaction databases that primarily operate as product-centered or opaque rule-based systems, SafeRx was designed as a substance-centric and provenance-aware infrastructure in which interaction entities remained linked to their originating evidence source and enrichment lineage.

The system architecture additionally maintained explicit separation between authoritative source-derived information and AI-assisted enrichment metadata, thereby preserving interpretability, provenance traceability, and clinical auditability throughout the entire processing pipeline.

SafeRx is publicly accessible through an open-access web-based interface “https://saferx.web.app (accessed on 11 May 2026)” designed to support transparent exploration of harmonized interaction, regulatory safety, lactation, and intravenous compatibility data for research, educational, and clinical decision-support purposes. SafeRx was implemented on a managed PostgreSQL 17 backend (Supabase) with a .NET 10/Entity Framework Core 10 application layer.

The resulting platform enables transparent exploration of harmonized drug–drug interactions, regulatory safety warnings, lactation-related risks, and intravenous compatibility data through a unified provenance-aware clinical interface. The infrastructure was designed to support medication-safety research, pharmacist-assisted interaction validation, and clinical decision-support workflows within a unified pharmaceutical knowledge environment. The detailed operational workflows implementing the architectural layers shown in Figure 1 are presented in Section 2.3, Section 2.4 and Section 2.5. These workflows describe the transformation of source-level pharmaceutical information into canonical substance entities and subsequently into harmonized interaction records that populate the final SafeRx knowledge infrastructure.

### 2.2. Data Sources and Knowledge Acquisition

SafeRx integrated pharmaceutical knowledge from multiple heterogeneous biomedical and regulatory data sources relevant to medication safety assessment and clinical interaction analysis.

Integrated data sources included Romanian Summary of Product Characteristics (RCP) documents, Danish drug–drug interaction repositories, OpenFDA boxed warning datasets, LactMed lactation safety records, and Stabilis intravenous compatibility databases, each contributing complementary regulatory, pharmacologic, and clinical safety information to the unified knowledge infrastructure.

Each integrated source contributed a distinct pharmacologic or regulatory knowledge domain, including interaction severity, contraindications, boxed warnings, reproductive safety information, intravenous compatibility data, mechanistic toxicity annotations, and clinical monitoring recommendations. All imported records retained explicit provenance metadata describing the originating database, extraction source, evidence lineage, and enrichment origin in order to preserve transparency and source-level traceability throughout the integration workflow.

This provenance-aware architecture enabled transparent differentiation between directly extracted authoritative data and AI-assisted enrichment layers.

### 2.3. Romanian RCP Processing Pipeline

Romanian RCP documents represented a major regulatory knowledge source within the SafeRx infrastructure. Unstructured regulatory documents were transformed into machine-readable pharmaceutical knowledge through a multi-stage extraction and normalization workflow. Within the overall architecture shown in Figure 1, the Romanian RCP workflow represented one of the principal source-ingestion pipelines responsible for generating structured regulatory safety data.

**Figure 2:**
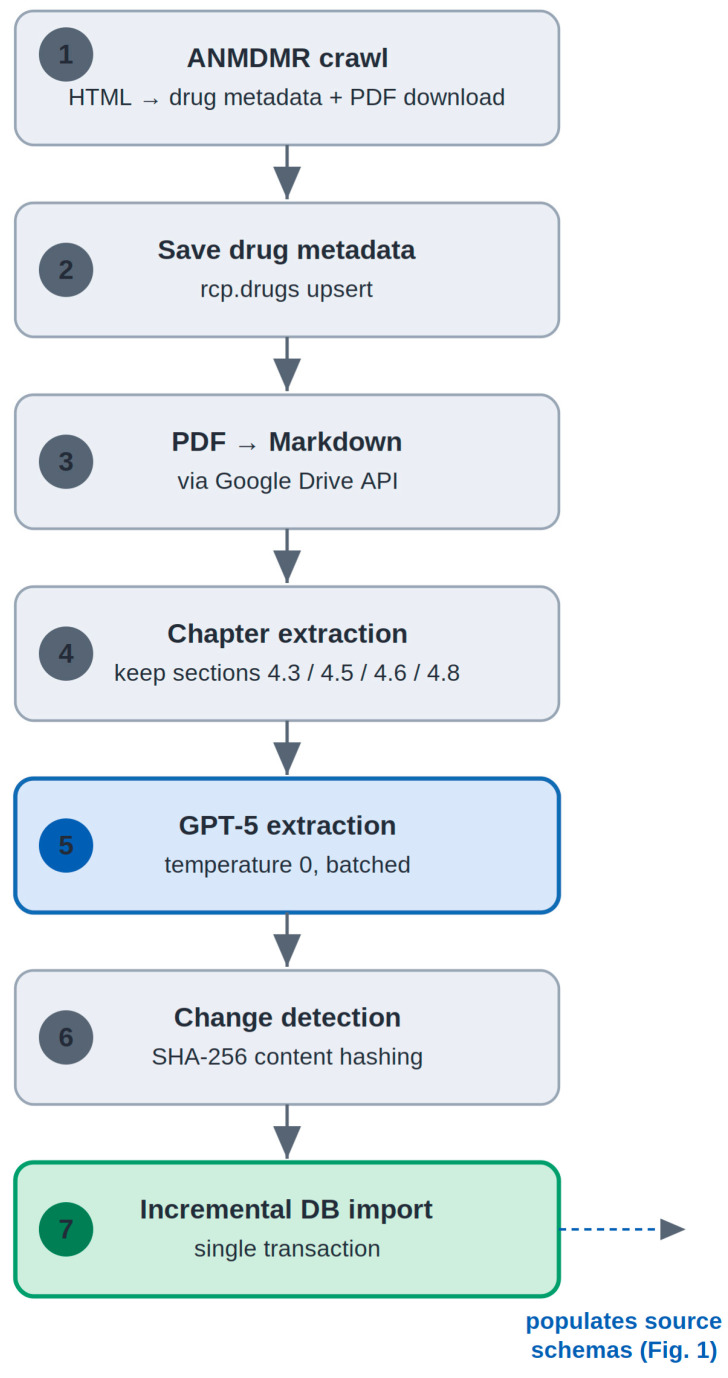
Romanian RCP extraction and normalization workflow. Seven-stage pipeline transforming unstructured Romanian RCP documents into structured pharmaceutical knowledge entities for downstream canonical normalization, harmonization, and integration within the SafeRx knowledge infrastructure. GPT, Generative Pre-trained Transformer; SHA-256 = Secure Hash Algorithm 256-bit.

The processing pipeline consisted of automated document acquisition, PDF-to-text conversion, Markdown transformation, section-level segmentation, interaction-focused content isolation, structured semantic extraction, canonical substance matching, enrichment procedures, and subsequent integration into the unified database infrastructure. The major stages of the Romanian RCP extraction and normalization workflow are summarized in Figure 2. The workflow comprised automated ANMDMR acquisition, PDF-to-Markdown conversion, extraction of clinically relevant safety sections (4.3, 4.5, 4.6, and 4.8 of each tested RCP), low-variance (temperature-zero) GPT-assisted structured extraction, SHA-256-based change detection, and incremental database integration. The resulting structured entities subsequently populated the interaction, contraindication, pregnancy, lactation, and adverse-reaction layers of the SafeRx knowledge infrastructure.

Interaction-relevant sections were identified and processed using structured GPT-assisted extraction workflows configured for low-variance, structured JSON-formatted outputs (sampling temperature set to zero). The extraction layer was designed to identify interacting substances, interaction severity classifications, contraindications, pharmacologic mechanisms, clinical consequences, and structured management recommendations from unstructured regulatory text sources.

To mitigate semantic anchoring and structural output errors during structured extraction, several layered safeguards were incorporated into the RCP processing pipeline. Decoding was performed with the sampling temperature fixed at zero for both the structured-extraction call and the substance-identity validation step, minimizing run-to-run variability. To reduce contextual anchoring and cross-section information bleed, each RCP document was structurally normalized and then restricted to the four clinically relevant RCP sections of the tested medicinal products (4.3, 4.5, 4.6, and 4.8) prior to extraction, while an upstream validity gate discarded non-regulatory documents that did not contain the expected RCP markers, ensuring the model never processed out-of-domain content. The extraction prompt constrained the model to the source text by requiring preservation of original wording, emission of fixed sentinel values for absent data, omission of undeterminable onset information, and restriction of every machine-readable field to a closed controlled vocabulary. Structural integrity was enforced after generation: responses that did not parse as valid JSON were rejected and never persisted, and a deterministic post-processing layer re-mapped out-of-vocabulary or mistyped values to the permitted enumeration or to a null value. Extraction was additionally content-addressed through SHA-256 hashing of the section-filtered input, ensuring idempotent reprocessing, after which all AI-extracted records were stored as provisional entries and routed to the pharmacist-supervised review workflow.

Extracted entities subsequently entered the canonical normalization and identity-resolution workflow described in Section 2.4, where heterogeneous substance representations originating from different regulatory, pharmacologic, and ontology-derived sources were reconciled into unified canonical master-substance records.

### 2.4. Canonical Substance Normalization and Identity Resolution

A central architectural component of SafeRx consisted of canonical substance normalization and cross-source pharmaceutical identity harmonization. This layer received structured entities generated by the source-specific extraction workflows and transformed them into interoperable canonical substance representations suitable for cross-source integration.

Heterogeneous pharmaceutical datasets frequently contained multilingual naming variations, trade names, formulation-specific variants, synonym inconsistencies, combination products, and source-dependent terminology, creating substantial challenges for cross-database interoperability and semantic harmonization.

To address these inconsistencies, SafeRx implemented a substance-centric master schema designed to harmonize pharmaceutical identifiers across heterogeneous datasets.

**Figure 3:**
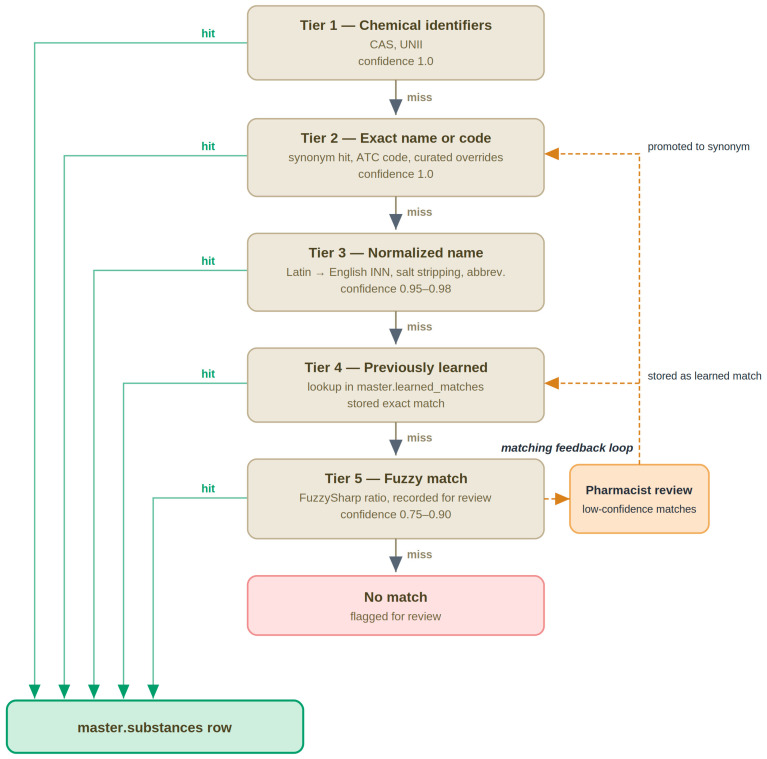
Multi-tier canonical substance identity-resolution cascade. Sequential identity-resolution workflow based on standardized identifiers, terminology harmonization, validated mappings, and semantic similarity, with unresolved entities routed for pharmacist-supervised review. CAS, Chemical Abstracts Service; INN, International Nonproprietary Names.

Canonical entities were harmonized using multiple standardized identifier systems, including International Nonproprietary Names (INN), DCI terminology, Unique Ingredient Identifiers (UNII), Chemical Abstracts Service (CAS) identifiers, Chemical Entities of Biological Interest (ChEBI) ontology mappings, PubChem identifiers, International Chemical Identifier Keys (InChIKeys), and Anatomical Therapeutic Chemical (ATC) classifications, enabling cross-source semantic interoperability and ontology-aware integration. The multi-tier identity-resolution cascade used for canonical substance matching is summarized in Figure 3. The workflow applies a hierarchical matching strategy in which authoritative identifier-based approaches are prioritized before lower-confidence terminology-based and probabilistic matching procedures. Subsequent matching tiers are evaluated only when preceding resolution layers fail to produce a canonical match, while unresolved entities are automatically flagged for pharmacist-supervised review and curation.

To improve robustness across heterogeneous sources, the identity-resolution layer incorporated explicit conflict-detection and reconciliation procedures. When multiple candidate matches were identified for the same substance, identifier-based evidence (e.g., CAS, UNII, InChIKey, PubChem, or ChEBI identifiers) was prioritized over terminology-based matches. Consequently, situations in which a name-based match conflicted with an identifier-derived match were resolved in favor of the identifier-supported canonical entity. Ambiguous or unresolved cases were not automatically merged and were instead routed to pharmacist-supervised review. This conservative strategy was intended to minimize incorrect canonical assignments while preserving traceability of reconciliation decisions.

Identity resolution operated through exact-match identifier lookups applied in a fixed precedence (InChIKey, CAS, UNII, PubChem, and ChEBI, followed by name-based matching), with probabilistic name matching used only as a terminal fallback. Name discrepancies between FDA UNII and ChEBI entries were therefore reconciled indirectly rather than through direct string comparison: a shared physical identifier (e.g., CAS or the full InChIKey) mapped both records to the same canonical substance irrespective of differing display names. For salts, esters, and hydrates, reconciliation was lexical rather than structure-based, with a curated list of pharmaceutical salt and hydrate suffixes removed prior to name-level matching (e.g., metformin hydrochloride → metformin), whereas stereochemical descriptors were handled only at the string level by removing leading optical-configuration prefixes (d-, l-, dl-, r-, s-, rs-) from Danish-source names. Because the full 27-character InChIKey was compared rather than the 14-character skeleton block, and because the ChEBI import retained only is_a and has_role relations without conjugate-acid/base, tautomer, enantiomer, or functional-parent relations or UNII parent hierarchies, a free base and its corresponding salt, as well as distinct stereoisomers of the same base molecule, were retained as separate canonical substances by design. This conservative strategy was adopted to avoid unintended conflation of substances that may differ in clinically relevant properties such as intravenous compatibility or formulation-specific handling.

The identity-resolution layer additionally incorporated synonym harmonization and canonical mapping propagation for supporting the interoperability between regulatory, pharmacologic, and ontology-derived datasets. This canonicalization strategy enabled semantic deduplication, cross-source interaction consolidation, ontology-aware linkage, and provenance-preserving aggregation of heterogeneous interaction records within the unified pharmaceutical knowledge graph. The canonical master-substance entities generated through this workflow subsequently served as the direct input for the interaction-consolidation and deduplication procedures described in Section 2.5.

### 2.5. Master Interaction Schema and Interaction Harmonization

Following normalization, interaction records were consolidated into a unified canonical interaction layer designed to represent substance-level interaction entities independently from product-specific redundancy.

The harmonization framework was designed to resolve bidirectional interaction duplication, synonym-derived redundancy, product-level repetition, and semantically overlapping interaction representations across heterogeneous pharmaceutical data sources.

**Figure 4:**
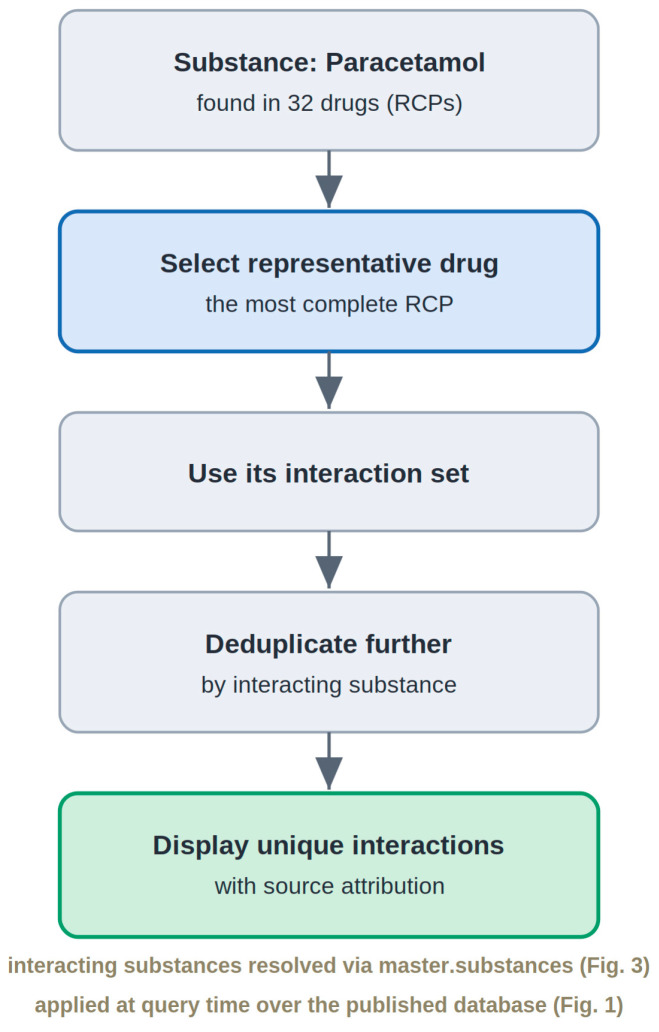
Canonical interaction deduplication and representative-source selection workflow. Product-level interaction records were consolidated into canonical interaction pairs while preserving source attribution and evidence provenance.

Instead of preserving redundant product combinations, SafeRx aggregated records into canonical interaction pairs while retaining source attribution and evidence provenance. For substances represented by multiple pharmaceutical products, a representative regulatory record with the most complete interaction documentation was preferentially selected prior to downstream enrichment and canonical interaction consolidation. The interaction deduplication and representative-source selection strategy used for canonical interaction consolidation is summarized in Figure 4, illustrating a specific example involving paracetamol. Representative regulatory records were selected based on interaction completeness and documentation density prior to downstream enrichment. Additional deduplication was subsequently performed at the interacting-substance level, enabling consolidation of semantically equivalent interaction records while preserving provenance metadata and source-level traceability.

The master interaction schema additionally supported cross-source interaction reconciliation, conflict-aware aggregation, canonical interaction-pair generation, and consolidated tier assignment workflows. Interaction provenance was preserved throughout the harmonization process, enabling transparent identification of source contribution, corroborating evidence layers, and enrichment origin for each integrated interaction entity. The resulting canonical interaction pairs subsequently entered the enrichment, prioritization, provenance aggregation, pharmacist-review, and publication layers summarized in Figure 1, thereby completing the end-to-end SafeRx knowledge integration workflow.

Conflict resolution within the interaction layer followed a provenance-preserving harmonization strategy. Source-specific interaction records were retained after normalization to the common canonical interaction framework, while severity representations originating from different source vocabularies were mapped to the unified SafeRx tier system. In cases where multiple sources contributed information for the same canonical interaction pair, reconciliation was performed using a safety-oriented approach that preserved all source attribution while prioritizing the most clinically severe classification for consolidated display and downstream decision-support workflows. Missing identifiers, orphan records, duplicate entities, and parser-generated artifacts were evaluated through dedicated quality-control procedures before removal or consolidation, thereby reducing the risk of information loss during harmonization.

### 2.6. AI-Assisted Clinical Enrichment Framework

The AI-assisted enrichment framework was designed to operate as a deterministic extraction and semantic structuring layer applied to interaction content already sourced from authoritative regulatory and clinical repositories, rather than as an autonomous interaction prediction system. Consequently, GPT-5-assisted workflows did not generate novel pharmacologic knowledge independently, but instead extracted, structured, and operationalized clinically relevant information originating from validated source documents, including Romanian regulatory product summaries, OpenFDA boxed warning datasets, and established pharmacologic repositories. The separation between authoritative source-derived content and AI-assisted enrichment metadata was explicitly maintained throughout the processing pipeline in order to preserve provenance transparency, interpretability, and clinical auditability of all generated annotations.

AI-assisted enrichment workflows combined semantic extraction, pharmacologic inference, rule-based propagation, mechanism-aware classification, and toxicity-oriented overlay integration to generate clinically interpretable interaction metadata. The enrichment framework produced structured annotations related to acute clinical relevance, onset timing, mechanistic interaction categories, semantic clinical effects, management recommendation fields, and never-event classifications. The system explicitly separated authoritative extracted content from AI-assisted enrichment metadata for preserving provenance transparency and interpretability.

Enrichment provenance tracking additionally differentiated GPT-derived semantic representations, propagated pharmacologic logic, mechanism-derived inference processes, and regulatory toxicity overlays for keeping the transparency regarding the origin and transformation pathway of derived clinical metadata.

All AI-assisted extraction and enrichment steps were executed through the Azure OpenAI Service using the official Azure OpenAI .NET SDK, with the OpenAI GPT-5 model (gpt-5-chat deployment). Primary extraction was implemented as a two-message chat completion, comprising a version-controlled system prompt that defined the extraction task and output format and a user message carrying only the chapter-filtered source text. The prompt used for data extraction is provided in Supplementary Material S1. The auxiliary steps (i.e., substance-name and ATC-name matching, acute-relevance tier classification, and Danish-to-English translation) used separate constrained prompts, with the tier-classification call also performed at a sampling temperature of zero and the translation helper using the service-default temperature. Temperature-zero decoding yields low-variance rather than bit-exact output, as the pipeline does not fix a decoding seed or enforce a server-side JSON schema, and the served model version is determined by the named Azure deployment rather than pinned in code. No automatic call-level retry was applied to the model calls. Automated quality-control checks verified that returned values conformed to the permitted enumerations and were complemented by a small set of expert-authored gold-standard fixtures asserting the expected structural output. A comprehensive held-out accuracy and inter-run agreement evaluation against an expert-adjudicated gold standard was not performed in the present study and is planned as part of the prospective clinical validation effort.

### 2.7. Temporal and Mechanistic Interaction Annotation

The enrichment framework implemented dedicated temporal and mechanistic interaction annotation workflows designed to improve clinical interpretability and actionability.

Temporal annotation pipelines classified interaction onset patterns into clinically relevant categories including rapid, delayed, and immediate onset interactions. Temporal classifications were generated using deterministic mechanism-aware inference, structured extraction logic, and pharmacologic propagation rules integrated within the enrichment framework.

Mechanistic annotation workflows harmonized pharmacodynamic, pharmacokinetic, toxicologic, and physicochemical interaction mechanisms across canonical interaction entities.

Annotated mechanism domains included bleeding risk, QT prolongation, respiratory depression, nephrotoxicity, hepatotoxicity, intravenous incompatibility, precipitation reactions, CYP-mediated metabolic interactions, protein-binding interactions, absorption-related interactions, excretion-related interactions, and additive pharmacodynamic effects.

### 2.8. Semantic Clinical Actionability Layer

To improve clinical usability, SafeRx transformed narrative interaction descriptions into structured semantic management objects. The semantic actionability framework generated structured representations of clinical effects, management recommendations, monitoring strategies, contraindication logic, and therapeutic precaution annotations in order to transform narrative interaction content into clinically actionable decision-support entities. These representations enabled interaction records to be displayed as clinically actionable decision-support entities rather than unstructured narrative text alone.

The recommendation architecture additionally supported downstream prioritization and tier-based interaction management workflows.

### 2.9. Consolidated Interaction Prioritization Framework

Following harmonization and enrichment, SafeRx generated a consolidated interaction layer integrating canonical interaction pairs, source-aware aggregation workflows, tier-based prioritization, and never-event classification logic within a unified operational interaction framework. Within SafeRx, a never-event designation refers to an interaction considered sufficiently hazardous that co-administration should generally be avoided because of the risk of severe, potentially irreversible, or life-threatening patient harm. Operational prioritization was implemented through a three-tier alerting framework: Tier 1 (hard-stop alerts requiring interruption of workflow), Tier 2 (interruptive alerts requiring explicit acknowledgment), and Tier 3 (informational or passive alerts displayed without workflow interruption). The prioritization framework implemented operational interaction stratification intended to support clinical decision-support workflows. Interaction tiers represented operational prioritization constructs incorporating interruptive alert handling, clinical relevance stratification, and escalation logic rather than functioning solely as conventional descriptive severity labels.

Each interaction record was classified into one of three operational clinical-priority categories reflecting the recommended response behavior within the decision-support environment. Tier 1 interactions represented immediate life-threatening or potentially irreversible-risk scenarios requiring hard-stop interruption logic, Tier 2 interactions represented clinically significant interactions requiring explicit acknowledgment prior to continuation, whereas Tier 3 interactions corresponded to informational, chronic, or lower-urgency interaction scenarios displayed through passive alerting mechanisms.

Tier assignment incorporated both GPT-assisted semantic classification workflows and deterministic rule-based enrichment procedures. A dedicated classification workflow evaluated interaction effect descriptions, management recommendations, and mechanistic annotations for generating harmonized prioritization labels and never-event annotations. To reduce redundant classification across equivalent pharmaceutical products, prioritization metadata could subsequently be propagated between interaction records sharing identical canonical substance pairs and interacting-substance relationships.

The prioritization layer additionally incorporated provenance-aware enrichment tracking mechanisms intended to preserve interpretability and auditability of derived prioritization metadata. The consolidated interaction framework further supported cross-source reconciliation workflows, harmonized best-tier assignment strategies, and provenance-retention mechanisms for canonical interaction prioritization across heterogeneous pharmaceutical data sources.

### 2.10. FDA Regulatory Safety Integration

SafeRx incorporated a dedicated regulatory safety layer derived from OpenFDA boxed warning datasets. The FDA integration workflow extracted and harmonized boxed warning annotations, toxicity-specific regulatory warnings, cardiovascular risk alerts, respiratory depression warnings, QT-related toxicity signals, bleeding-related toxicity alerts, hepatotoxicity annotations, and nephrotoxicity signals derived from OpenFDA regulatory datasets.

Regulatory warning entities were subsequently mapped to canonical master substances and integrated into the unified interaction knowledge graph. This integration enabled regulatory toxicity overlays to complement the canonical interaction enrichment framework.

### 2.11. Lactation Safety Integration

To support reproductive and breastfeeding-related medication safety assessment, SafeRx incorporated LactMed-derived lactation safety information into the unified pharmaceutical knowledge infrastructure.

Integrated lactation records included maternal exposure considerations, infant exposure information, breastfeeding-related safety effects, therapeutic alternatives, and milk-related pharmacokinetic annotations relevant to medication safety assessment during lactation.

The LactMed integration layer additionally incorporated specialized reproductive hazard annotations related to cytotoxic agents and radioactive compounds associated with breastfeeding-related medication safety risks.

Lactation-related entities were harmonized to canonical substance identifiers before integration into the unified interaction layer.

### 2.12. Intravenous Compatibility Integration

SafeRx additionally incorporated a dedicated intravenous compatibility layer derived from the Stabilis database.

The compatibility framework integrated compatible and incompatible intravenous medication pairs, concentration-dependent compatibility data, solvent-specific compatibility information, precipitation-related incompatibility annotations, evidence references, and physicochemical compatibility notes derived from the Stabilis database.

Compatibility entities were normalized through the canonical substance harmonization pipeline prior to integration into the consolidated pharmaceutical knowledge graph.

This architecture enabled SafeRx to support compatibility-aware medication assessment in addition to conventional drug–drug interaction analysis.

### 2.13. Pharmacist Validation and Clinical Governance Workflow

To support clinical reliability, expert oversight, and longitudinal quality assurance, SafeRx incorporated a pharmacist-supervised validation and governance workflow integrated directly into the platform infrastructure.

Newly generated, enriched, or updated interaction entries were initially routed to a pending-review environment prior to incorporation into the validated interaction layer. Authorized pharmacists could review interacting substances, interaction summaries, prioritization tiers, mechanistic annotations, clinical management recommendations, and enrichment provenance metadata through a structured moderation interface supporting approval, rejection, modification, and collaborative review workflows.

The validation architecture additionally incorporated invitation-based pharmacist onboarding, enabling controlled expert participation within the review and curation process. Human review actions were integrated with provenance-aware auditability mechanisms designed to preserve traceability, reviewer accountability, and longitudinal documentation of interaction-level modifications and validation decisions.

This human-in-the-loop governance framework was designed to combine automated pharmaceutical knowledge extraction and enrichment with pharmacist-supervised clinical verification while preserving transparency, interpretability, and regulatory traceability throughout the interaction validation pipeline.

To assess the accuracy and clinical reliability of the canonical harmonization and enrichment workflows, a structured expert validation procedure was conducted prior to platform deployment. A sample of 200 canonical substances, selected by random sampling without replacement from the full canonical corpus (*n* = 2190), was independently reviewed by three clinical pharmacy specialists and researchers (A.F.R, A.R. and D.M.T.) with expertise in pharmaceutical sciences and medication safety. The validation procedure evaluated canonical identity resolution accuracy, enrichment annotation correctness, tier assignment plausibility, and mechanistic annotation consistency. Reviewer evaluation confirmed accurate canonical identity resolution for 91% of assessed substances, with enrichment annotations and tier assignments considered clinically plausible and consistent with source documentation. The validation procedure was conducted through structured consensus review, with the corrected nomenclature for substances identified as incorrectly named established through collaborative discussion prior to platform deployment.

Each substance was independently classified by all three reviewers as correctly or incorrectly named, with the assigned canonical name evaluated for conformance to standard International Nonproprietary Name (INN) nomenclature and standardized chemical identifiers (CAS, UNII, ChEBI, InChIKey) used as corroborating references for substance identity. The three reviewers were fully concordant in their independent classifications (observed agreement 100%; Fleiss’ κ = 1.00), unanimously identifying the same 18 of 200 substances (9%) as carrying non-standard name variants (e.g., misspelled or phonetically transliterated forms such as “azelaztine”, “metadone”, and “warfaryn sodium”). For these 18 substances, the corrected canonical name (azelastine, methadone, and warfarin sodium, respectively) was established through collaborative discussion and recorded in the master-substance table; the complete list of reviewed canonical substances is provided in Supplementary Material S2. The reported identity-resolution accuracy of 91% (182 of 200) corresponds to the proportion of canonical substance names conforming to standard nomenclature without correction.

Moreover, to quantify the accuracy of the GPT-derived acute-relevance tier assignments, a dedicated blinded validation was conducted. A stratified random sample of 90 interactions (30 per Tier 1, Tier 2, and Tier 3) was drawn from the GPT-classified RCP enrichment records and independently re-classified into Tier 1, 2, or 3 by three clinical pharmacy specialists (A.F.R., A.R., D.M.T.), blinded to the system-assigned tier and to one another, using only the interaction description (index substance, interacting substance/class, effect, recommendation, and mechanism). Agreement with the system tier was quantified as percentage agreement and linear-weighted Cohen’s κ, inter-rater reliability as Fleiss’ κ with bootstrap 95% confidence intervals, and the distribution of assignments as a 3 × 3 confusion matrix. Discordant cases were examined for direction (over- versus under-triage) and clinical pattern.

The publicly accessible architecture additionally enables continuous post-deployment expert review, longitudinal refinement of interaction annotations, and transparent validation of newly integrated pharmaceutical safety information. SafeRx is freely accessible through a public web-based interface designed to support transparent exploration, validation, and review of harmonized medication-safety data for research and advisory clinical decision-support purposes.

## 3. Results

### 3.1. Canonical Pharmaceutical Knowledge Corpus

The SafeRx platform integrated a unified pharmaceutical knowledge corpus centered on canonical substance-level harmonization. The core canonical layer contained 2190 distinct master substances.

A large-scale identifier harmonization layer was subsequently constructed using multiple standardized chemical and regulatory identifier systems. In total, 9463 validated identifier mappings were linked to canonical substances, corresponding to 92.05% canonical coverage (2016 mapped substances of 2190 total canonical substances). A comprehensive overview of identifier mapping categories, mapping distributions, and provenance-aware integration sources is provided in Table 1.

Master-registry mappings refer to identifier mappings derived from the internal canonical master-substance layer of SafeRx (source = master) rather than from external databases. Each mapping linked a canonical master substance to a standardized identifier type and value while preserving provenance source and confidence metadata. Confidence values were recorded on a 0–1 scale. Higher confidence values reflected stronger identifier-level agreement within the harmonization framework, whereas lower confidence values were reserved for a small subset of curated CAS and UNII mappings requiring additional harmonization steps. In the final harmonization layer, 9012 mappings (95.23%) had confidence 1.00, 433 mappings (4.58%) had confidence 0.95, and 18 mappings (0.19%) had confidence 0.90. All PubChem, InChIKey, and ChEBI mappings were assigned confidence 1.00, whereas reduced-confidence mappings occurred exclusively among CAS and UNII identifiers.

The provenance-aware harmonization layer was dominated by FDA UNII-derived mappings (53.0%), followed by internally curated canonical mappings (29.5%) and ChEBI ontology integration (16.1%). The predominance of maximal-confidence mappings indicates that the identifier harmonization framework was largely composed of high-confidence canonical associations. These findings indicate large-scale identifier-level harmonization across heterogeneous pharmaceutical and biomedical resources, supporting semantic interoperability at the level of substance identity.

### 3.2. Multi-Source Interaction Knowledge Integration

The interaction knowledge layer contained 10,484 canonical interaction records integrated from multiple regulatory and clinical data sources.

Canonical pair normalization reduced the interaction corpus from 10,484 raw interaction rows to 9932 unique canonical interaction pairs, corresponding to a 5.27% duplicate reduction rate after consolidation of bidirectional and semantically redundant interaction entities.

**Figure 5:**
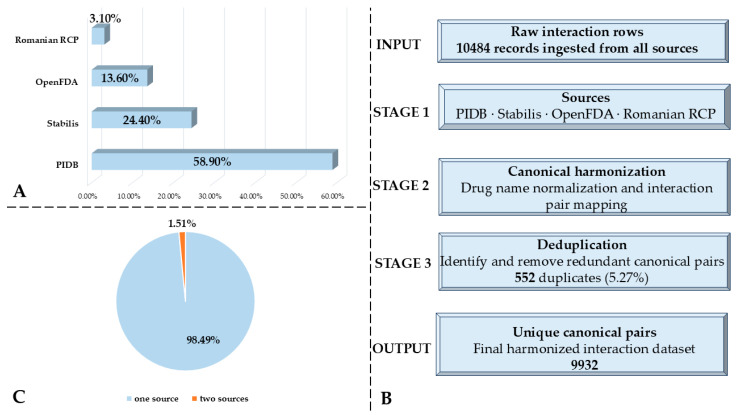
Interaction harmonization and canonical consolidation workflow. (**A**) Relative contribution of interaction records across integrated data sources. (**B**) Canonical harmonization and deduplication workflow used to generate the final unified interaction dataset. (**C**) Cross-source corroboration analysis showing the proportion of interactions supported by single versus multiple integrated sources.

Cross-source corroboration analysis demonstrated that most interaction pairs were supported by a single specialized source (9782 pairs, constituting 98.49% of the total), reflecting complementary rather than redundant integration across heterogeneous medication safety domains. These findings indicate that most integrated medication-safety information originated from complementary rather than overlapping evidence domains. Figure 5 illustrates the interaction harmonization and canonical consolidation workflow used to generate the final unified interaction dataset from heterogeneous regulatory and clinical interaction sources. Only 150 of 9932 canonical interaction pairs (1.51%) were supported by multiple integrated sources, indicating that direct cross-source conflicts were uncommon within the consolidated dataset. For these multi-source interaction pairs, provenance metadata from all contributing sources was retained, and harmonized prioritization was generated through the conflict-resolution framework described in Section 2.5.

### 3.3. Clinical Interaction Enrichment Layer

A dedicated clinical enrichment framework was applied to the canonical interaction layer. In total, 6403 enriched interaction records were generated through semantic extraction, pharmacologic inference, and rule-based propagation.

#### 3.3.1. Temporal Interaction Enrichment

Among records containing temporal annotations, rapid-onset interactions predominated, accounting for 76.56% of annotated pairs (*n* = 1107). Delayed-onset interactions represented 23.03% of this subset (*n* = 333), while immediate-onset interactions were rare, comprising only 0.41% of annotated records (*n* = 6).

#### 3.3.2. Acute Clinical Relevance Prioritization

The enrichment framework initially generated record-level acute clinical relevance annotations prior to canonical consolidation and conflict-aware prioritization. These enrichment-stage tiers represented preliminary semantic prioritization signals applied at the interaction-record level.

Within the enrichment layer, 290 interaction records (4.53%) were initially classified as never-events prior to cross-source consolidation and canonical propagation, representing highly critical interaction combinations requiring strict avoidance or maximal clinical caution.

Tier 1 interactions represented the highest-priority interaction category associated with potentially life-threatening or irreversible clinical outcomes and accounted for 893 interaction records (14.50%). Tier 2 interactions represented clinically significant interruptive alerts requiring active acknowledgment and included 1712 interaction records (27.81%). Tier 3 interactions corresponded to lower-urgency, chronic, or informational interaction scenarios displayed through passive prioritization logic and represented the largest interaction category with 3552 interaction records (57.69%).

**Figure 6:**
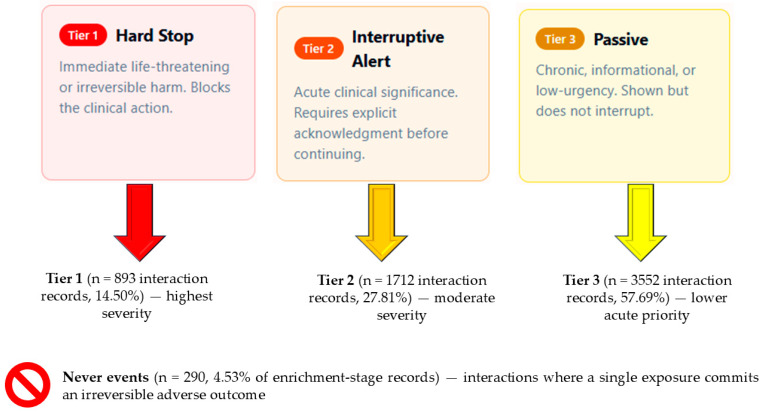
Distribution of enrichment-stage acute clinical relevance tiers and never-event interaction annotations.

Figure 6 summarizes the enrichment-stage operational prioritization framework together with the distribution of Tier 1, Tier 2, Tier 3, and never-event interaction annotations generated during semantic enrichment.

#### 3.3.3. Mechanistic Interaction Annotation

Mechanistic annotations were successfully harmonized across canonical interaction entities. The predominant mechanistic categories are summarized in Table 2.

Additional pharmacokinetic and pharmacodynamic mechanism classes included CYP-mediated inhibition and induction, absorption decrease, and excretion decrease. Furthermore, the analysis incorporated protein-binding interactions, additive pharmacodynamic effects, hepatotoxicity, nephrotoxicity, hyperkalemia, and neuromuscular blockade.

#### 3.3.4. Structured Clinical Recommendation Fields

Structured recommendation fields were available for 8000 interaction records (76.3%), enabling transformation of narrative interaction text into structured, machine-readable clinical management fields.

Furthermore, structured semantic effect descriptions were available for 10,334 interactions (98.56%), indicating high semantic completeness of the harmonized interaction corpus.

#### 3.3.5. Tier Assignment Provenance

Among the 6157-tiered RCP interaction records, tier-assignment provenance was predominantly GPT-derived: 5368 records (87.19%) were assigned by the language model, 454 (7.37%) were tier-propagated to INN-equivalent pairs, and the remainder were set by deterministic rule-based safety overrides. The remaining assignments were distributed across highly specific clinical and regulatory safety guidelines, including lactmed_cytotoxic (46 counts, 0.75%) and various FDA-specified risk categories such as cardiovascular, nephrological, respiratory, and bleeding risks, each contributing less than 1% to the total distribution. Notably, the most safety-critical tier classifications were not determined by the language model. Within the RCP enrichment layer, 315 tier assignments (5.1%) were set by deterministic rule-based safety overrides (FDA boxed-warning toxicity classes, LactMed cytotoxic status, Stabilis intravenous incompatibility), and the same scaffold was applied more extensively in the Danish (PIDB) layer, where 2431 of 6430 assignments (37.8%) were rule-based rather than GPT-derived. A deterministic never-event classification additionally flagged 2411 consolidated pairs (24.28%) independently of the model.

#### 3.3.6. Validation of GPT-Derived Tier Assignments

Across the 90 classified interactions evaluated, agreement with the system-assigned tier was 96.7% (87/90) for each of the three reviewers, and the reviewer majority vote agreed with the system tier in 98.9% of cases (89/90; 95% CI 96.7–100.0). Inter-rater reliability among the three pharmacists was high (Fleiss’ κ = 0.91, 95% CI 0.84–0.97; full three-way concordance on 82/90 items), and the mean reviewer-versus-system linear-weighted Cohen’s κ was 0.96. The 3 × 3 confusion matrix contained a single discordant case (1/90): one interaction assigned Tier 2 by the system, flurbiprofen with other non-steroidal anti-inflammatory drugs, was rated Tier 1 by the reviewer majority, representing an under-triage at the Tier 1/Tier 2 boundary. No interaction was misclassified across non-adjacent tiers. For the highest-severity category, the system reproduced 30 of the 31 interactions judged Tier 1 by the reviewer majority (sensitivity 96.8%) with no false-positive Tier 1 assignments (precision 100%). The complete re-classification dataset is provided in Supplementary Material S3.

### 3.4. Final Consolidated Interaction Layer

The final consolidated interaction summary layer contained 9932 consolidated canonical interaction pairs after duplicate reduction, cross-source reconciliation, and conflict-aware prioritization. Unlike enrichment-stage annotations, the final consolidated layer reflects conflict-aware canonical prioritization after duplicate resolution, propagation, and cross-source reconciliation. Table 3 summarizes the final consolidated interaction-tier architecture generated after duplicate reduction, propagation, and cross-source harmonization.

The proportion of Tier 1 pairs in the final consolidated layer (4630 pairs, 46.86%) is higher than at the enrichment stage (893 records, 14.50%). However, these two values are computed on different populations and should not be interpreted as a direct escalation effect. The enrichment-stage figure reflects RCP-derived interaction records tiered before cross-source consolidation, whereas the consolidated figure spans all integrated sources. The higher consolidated fraction is therefore primarily a source-composition effect: the consolidated layer additionally incorporates specialized sources that contribute predominantly high-severity records, most notably OpenFDA regulatory boxed-warning and toxicity annotations and Stabilis intravenous-incompatibility records, which were absent from the RCP-only enrichment figure. Consistent with the predominantly single-source corroboration of consolidated pairs (98.49%; Figure 5C), cross-source best-tier escalation can apply only to the small minority of pairs supported by more than one source and therefore contributes secondarily; its role is to preserve a genuine high-priority signal present in any contributing record rather than allowing it to be diluted during canonical deduplication. The elevated Tier 1 fraction thus reflects chiefly the inclusion of inherently high-acuity specialized safety domains rather than escalation across records, while the conservative best-tier policy continues to favor sensitivity over alert minimization.

The final prioritization framework resulted in substantial enrichment of high-priority canonical interaction pairs within the consolidated interaction graph. This consolidation strategy increased the representation of high-priority (Tier 1) interaction pairs within the final canonical interaction graph. Following canonical consolidation, propagation, and conflict-aware prioritization, 2411 canonical interaction pairs (24.28%) were ultimately classified as never-events in the final summary layer.

### 3.5. Specialized Safety Integration Layers

The platform incorporated multiple specialized safety enrichment layers derived from FDA, LactMed, and Stabilis datasets to enhance high-risk medication safety intelligence across complementary clinical domains.

The FDA regulatory safety layer contained 514 boxed warning records, of which 378 entries (73.54%) were successfully harmonized to canonical master substances, corresponding to 302 unique mapped substances. The predominant warning domains included cardiovascular toxicity, bleeding risk, respiratory depression, hepatotoxicity, QT prolongation, and nephrotoxicity.

The LactMed-derived lactation safety layerincorporated 1895 breastfeeding-related safety records, with 1428 entries (75.36%) successfully harmonized to canonical master substances. Additionally, the dataset identified cytotoxic and radioactive medication records relevant to breastfeeding-associated medication safety assessment.

The Stabilis-derived intravenous compatibility layer incorporated 9088 IV compatibility records integrated directly into the canonical substance interaction framework. Canonical substance harmonization was achieved for 9087 of 9088 IV compatibility pairs (99.99%), indicating near-complete identifier-level linkage between the IV compatibility data and the canonical substance graph. The single unmatched pair involved gelatin and vancomycin hydrochloride, classified by Stabilis as an incompatible admixture associated with immediate precipitation. While vancomycin hydrochloride was successfully resolved to the canonical substance vancomycin, gelatin lacked a corresponding canonical mapping and therefore could not be linked to a master-substance identifier. Consequently, this isolated source-level record was excluded from canonical compatibility aggregation.

**Figure 7:**
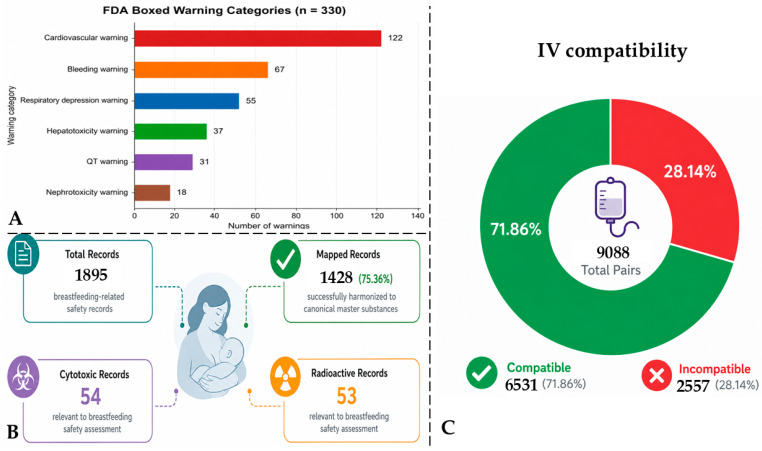
Specialized Safety Integration Layers. (**A**) Distribution of FDA boxed warning categories across harmonized regulatory safety records. (**B**) Overview of the LactMed-derived lactation safety enrichment layer, including mapped, cytotoxic, and radioactive records. (**C**) Distribution of compatible and incompatible intravenous compatibility pairs integrated from the Stabilis dataset.

Figure 7 summarizes the specialized safety integration layers incorporated into the platform, including FDA boxed warning domains, LactMed lactation safety enrichment, and intravenous compatibility integration.

### 3.6. Pharmacist-Supervised Validation Environment

SafeRx incorporated a pharmacist-supervised validation environment designed to support expert review of generated and enriched interaction records prior to publication within the validated interaction layer.

The dashboard interface provided a centralized pending-review workflow displaying interaction summaries together with severity tiers, mechanistic annotations, clinical management recommendations, and enrichment metadata. Pharmacists could subsequently approve, reject, or modify interaction entries through structured moderation actions integrated directly into the platform interface.

The review workflow additionally supported invitation-based pharmacist onboarding and collaborative expert participation within the validation process. This human-in-the-loop validation architecture provided an additional layer of clinical oversight for interaction curation, quality assurance, and longitudinal refinement of medication-safety annotations.

Every review action was recorded within an append-only audit log containing reviewer identity, moderation decisions, before-and-after record snapshots, and structured documentation of modified fields, thereby supporting regulatory traceability, reviewer accountability, and provenance-aware validation governance.

**Figure 8:**
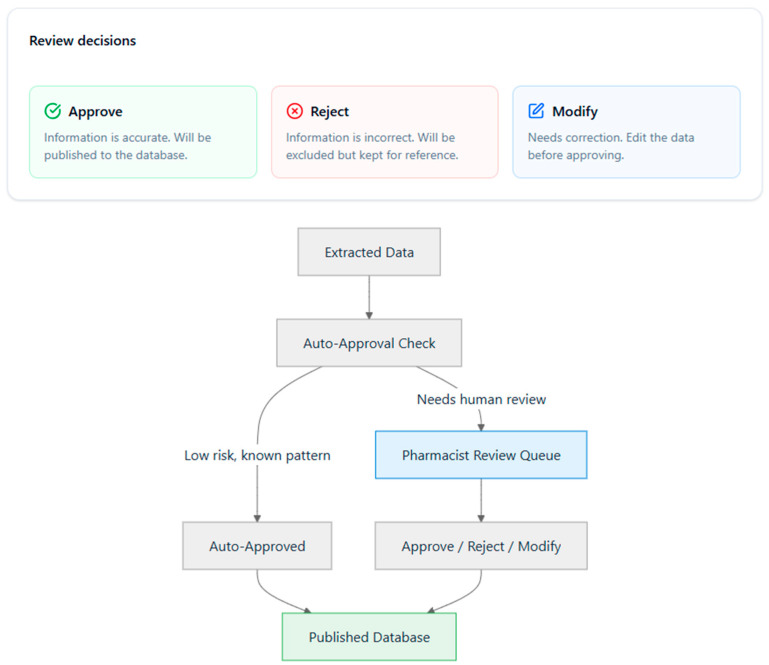
Pharmacist-supervised validation and moderation workflow.

Figure 8 demonstrates the pharmacist-supervised validation and moderation workflow used for structured review, approval, rejection, modification, and quality-control oversight of enriched interaction records prior to incorporation into the validated interaction layer.

In addition to the platform-level validation workflow, pharmacist-supervised validation generated two concrete validation outputs. Firstly, 200 canonical substance records were independently reviewed by three clinical pharmacy specialists. Accurate canonical identity resolution was confirmed for 182 of 200 substances (91%), while 18 substances (9%) required correction because of non-standard nomenclature, misspellings, or phonetically transliterated forms. The corrected records included examples such as azelaztine → azelastine, metadone → methadone, and warfaryn → warfarin. Inter-rater concordance for the identification of incorrectly named substances was complete (observed agreement 100%; Fleiss’ κ = 1.00).

Secondly, a blinded validation of 90 GPT-derived tier assignments demonstrated high agreement with the system tier: each reviewer agreed with 87 of 90 assignments (96.7%), majority-vote agreement was 89 of 90 (98.9%), and inter-rater reliability was very high (Fleiss’ κ = 0.91). The only discordant case involved flurbiprofen with other non-steroidal anti-inflammatory drugs, where reviewers escalated the system-assigned Tier 2 classification to Tier 1. Thus, the main pharmacist-identified issues were non-standard canonical nomenclature and one boundary-level tier under-triage case.

### 3.7. Representative Platform Workflow Example

To illustrate the operational functionality of the SafeRx platform at the substance level, phenytoin (CAS: 57-41-0; ATC: N03AB02) was selected as a representative high-risk medication example due to its narrow therapeutic index, clinically significant pharmacokinetic interaction profile, and intravenous administration complexity. Within the SafeRx substance browser, phenytoin was represented as a canonical entity harmonized across multilingual synonyms (fenitoina, phenytoine, phenytoinum) and linked to standardized identifiers, including CAS 57-41-0 and the ChEBI ontology entry CHEBI:8107. The Romanian RCP-linked commercial formulation PHENHYDAN 250 mg/5 mL (N03AB02) was additionally associated with the canonical substance record, demonstrating the platform’s ability to bridge product-level regulatory information with substance-centered knowledge harmonization.

The substance-level interface integrated regulatory warnings, interaction prioritization, reproductive safety information, and intravenous compatibility data through a unified substance-level clinical interface. An FDA boxed warning related to cardiovascular toxicity during rapid intravenous phenytoin administration was displayed directly within the overview layer together with QT prolongation and cardiovascular risk annotations, thereby enabling direct integration of regulatory safety overlays within the substance-level workflow.

The interaction layer identified 237 interaction records associated with phenytoin, including 219 interaction records classified as Tier 1 high-priority alerts. Interaction records were operationally stratified into tier-based clinical relevance groups, including Tier 1 hard-stop alerts and Tier 2 interruptive alerts. Structured interaction entries incorporated recommendation fields, mechanism annotations, provenance-aware source attribution, and never-event labeling alongside interaction prioritization data.

Representative clinically relevant interaction patterns included QT prolongation-associated combinations, CYP-related pharmacokinetic interactions, and physicochemical incompatibility alerts. Mechanism-aware annotations included QT prolongation and intravenous incompatibility categories, displayed together with structured management recommendations and source-linked evidence layers.

The specialized intravenous compatibility module displayed 63 compatibility records associated with phenytoin, including 55 incompatible combinations and 8 compatible combinations. Representative incompatibility patterns included immediate precipitation, immediate turbidity, and delayed precipitation phenomena observed after defined administration intervals. Concentration-dependent compatibility annotations were additionally available for selected intravenous combinations.

The platform further integrated LactMed-derived lactation safety information within the consolidated substance-level environment, including breastfeeding safety summaries, infant exposure considerations, and alternative therapeutic options. This representative workflow illustrates how SafeRx consolidates heterogeneous regulatory, pharmacologic, reproductive, and physicochemical medication-safety information into a unified multimodal clinical decision-support environment.

**Figure 9:**
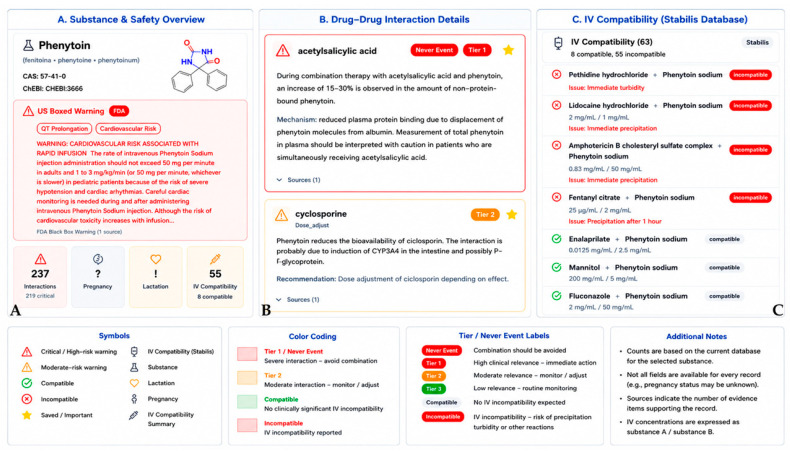
Representative SafeRx multimodal clinical workflow for phenytoin. (**A**) Substance-level overview integrating canonical identifiers, regulatory safety information, interaction burden, reproductive safety indicators, and intravenous compatibility summaries. (**B**) Tier-based interaction prioritization interface. (**C**) Intravenous compatibility module displaying compatibility outcomes and administration-safety information.

Figure 9 further illustrates the simultaneous integration of canonical substance harmonization, regulatory safety overlays, tier-based interaction prioritization, and concentration-dependent intravenous compatibility annotations within a unified clinical decision-support interface. Panel A demonstrates the integration of canonical identifiers with regulatory safety overlays, interaction burden metrics, reproductive safety information, and intravenous compatibility summaries. Panel B illustrates the prioritization layer, including tier-based alert stratification, structured recommendations, source attribution, and never-event annotations. Panel C presents concentration-dependent intravenous compatibility information derived from the Stabilis integration layer, including precipitation- and turbidity-related observations relevant to administration safety.

### 3.8. System Performance, Scalability, and Computational Characteristics

The consolidated platform was deployed on a managed PostgreSQL 17 backend (Supabase) with a .NET 10/Entity Framework Core 10 (Npgsql) application layer, and the database schema was reproducible from 56 version-controlled migrations. The integrated database comprised 18 schemas, 120 tables, and 372 indexes, stored approximately 1.04 million rows, and occupied 353 MB (Table 4). Computational cost was bounded by the incremental, content-hash-gated synchronization pipeline described in Section 2.3, together with per-active-ingredient extraction de-duplication, configurable concurrency limits (concurrent model calls, document conversions, and an inter-request crawl delay), and batched database operations. Query performance was supported by precomputed materialized views—rcp.search_index (40,057 rows) and master.interaction_summary (9932 pairs)—indexed with a trigram GIN index (idx_search_index_term_trgm) and a B-tree prefix index (idx_search_index_term_prefix), and refreshed concurrently (non-blocking) off-peak daily (03:00 UTC) via pg_cron. Across their recorded execution history, the scheduled refresh jobs completed reliably: refresh of rcp.search_index completed in a mean of 2.4 s (range 1.6–4.3 s; *n* = 158 runs) and refresh of atc.navigation_tree in a mean of 0.6 s (range 0.3–0.8 s; *n* = 172 runs), with all recorded runs succeeding; these durations refer to database-side materialized-view refresh jobs only and not to the full extraction–normalization–consolidation pipeline or to application-level performance. Representative database-level query plans obtained through EXPLAIN ANALYZE returned a ranked search over the materialized search index in 171.6 ms and a complete substance-detail lookup in 12.2 ms (Table 4); these values represent single-query PostgreSQL execution times and should not be interpreted as application-level latency or throughput. End-to-end pipeline wall-clock time and overall throughput on a fixed reference machine were not formally benchmarked in the present study.

## 4. Discussion

The present study describes the development and large-scale implementation of SafeRx, a substance-centered pharmaceutical knowledge integration and clinical decision-support platform designed to consolidate heterogeneous medication-safety resources within a unified canonical framework. The resulting infrastructure integrated drug–drug interaction data, regulatory toxicity overlays, LactMed-derived reproductive safety information, intravenous compatibility records, semantic enrichment layers, and pharmacist-supervised validation workflows into a harmonized multimodal medication-safety environment. The findings demonstrate the feasibility of constructing an interoperable and provenance-aware pharmaceutical knowledge architecture capable of operationally integrating multiple fragmented medication-safety domains within a single clinically navigable framework.

One of the most important contributions of the platform resides in its canonical harmonization architecture. Medication-safety datasets frequently originate from heterogeneous regulatory, pharmacologic, and physicochemical repositories using inconsistent naming systems, multilingual terminology, and product-centered identifiers. The canonical substance-resolution workflow enabled harmonization across multilingual synonyms, ontology-linked identifiers, and heterogeneous pharmaceutical naming conventions while preserving provenance-aware linkage throughout integration. The harmonization workflow extended beyond identifier normalization to enable semantic deduplication, bidirectional interaction reconciliation, and conflict-aware canonical pair generation within the consolidated interaction graph.

Large-scale terminology harmonization studies have similarly emphasized the interoperability challenges associated with integrating heterogeneous drug vocabularies into standardized pharmacologic knowledge environments. Interoperability analyses have shown that semantic inconsistencies, multilingual terminology variation, and divergent dose-form representations substantially complicate cross-platform medication-data integration, and that fine-grained reconciliation requires hierarchical refinement workflows incorporating strength, formulation, and branded-product relationships within ontology-aware architectures [13]. The SafeRx architecture reduced several of these limitations through multilingual synonym reconciliation, ontology-linked normalization, and provenance-aware canonical pair generation across the 9932 consolidated interaction pairs.

Additional interoperability studies reported that major vocabulary repositories exhibit substantial discrepancies in concept availability and ingredient-level mappings despite relying on widely adopted standards such as ATC and RxNorm, directly influencing CDS-oriented implementation within real-world EHR environments [28]. Hochheiser et al. highlighted that the absence of universally adopted semantic models complicates the exchange and computable interpretation of medication-safety information, proposing a minimal information model incorporating mechanistic annotations, contextual modifiers, and evidence attribution to improve semantic interoperability [29]. The SafeRx platform reduced several of these interoperability gaps through ontology-aware harmonization, conflict-aware reconciliation, and provenance-preserving semantic structuring across heterogeneous evidence sources.

Recent pharmacist-oriented semantic infrastructures have emphasized the importance of integrating heterogeneous pharmaceutical resources into interoperable and clinically navigable knowledge environments. Pharmacist-oriented semantic infrastructure studies have described how fragmented medication-information ecosystems force pharmacists to rely on multiple disconnected resources with inconsistent coverage and limited interoperability, and that existing biomedical knowledge graphs often remain insufficiently aligned with real-world pharmacist workflows and clinically actionable tasks [17]. The SafeRx framework similarly incorporated canonical harmonization, provenance-aware aggregation, and multimodal medication-safety integration to reduce fragmentation across regulatory, pharmacologic, reproductive-safety, and physicochemical datasets, consistent with the observation that clinically usable infrastructures require not only data integration but also semantically structured and operationally interpretable knowledge representations [17,30].

The predominance of single-source corroborated interaction pairs within the final consolidated interaction layer reflects the complementary rather than redundant structure of specialized medication-safety repositories, where regulatory toxicity annotations, reproductive safety information, intravenous compatibility records, and pharmacologic interaction datasets are commonly maintained in isolated operational silos. Consequently, integrating heterogeneous resources into a unified canonical architecture provides a broader medication-safety representation than isolated interaction-checking systems relying on single-domain repositories. Provenance-aware linkage preservation throughout the harmonization workflow additionally supports source-level transparency and traceability, increasingly recognized as essential requirements for explainable biomedical informatics infrastructures.

Pharmacovigilance-oriented standardization studies have also demonstrated the impact of heterogeneous medication nomenclature on data quality and signal-detection reliability. Chalabianloo et al. reported that ontology-linked normalization and RxNorm-centered harmonization substantially improved aggregation consistency and detection of previously diluted adverse-event associations, emphasizing that semantic standardization itself may directly improve interpretability and pharmacovigilance utility [31]. Previous comparative evaluations of commercial DDI screening systems have further demonstrated substantial variability in interaction detection accuracy, severity classification, and monograph completeness, with no evaluated platform considered fully sufficient as a standalone clinical solution [32]. These findings support the architectural rationale of SafeRx, where heterogeneous resources were intentionally consolidated within a provenance-aware canonical environment supported by structured prioritization layers and pharmacist-supervised validation.

A central component of the platform was the semantic enrichment framework designed to operationalize narrative interaction descriptions into structured clinically actionable representations. Traditional interaction repositories expose interaction data primarily as free-text descriptions that remain difficult to operationalize within structured CDS systems. The SafeRx enrichment architecture generated operational clinical tiers, recommendation fields, temporal annotations, mechanistic categories, and semantic prioritization layers while maintaining source attribution and enrichment provenance. Hsu et al. demonstrated the feasibility of NLP-based pipelines for structured information extraction from regulatory documents, while highlighting key challenges including synonym variability, inconsistent terminology, and heterogeneous document structures [33], challenges addressed through the SafeRx document processing and section-aware extraction workflows.

The tier-based prioritization process constituted a major operational constituent of the platform. Graded clinical relevance categories (i.e., Tier 1 hard-stop interactions, Tier 2 interruptive alerts, and lower-priority informational layers) were incorporated to address alert fatigue, a well-documented limitation of conventional CDS systems. Felisberto et al. reported that approximately 90% of DDI alerts are overridden by prescribers, with excessive low-priority alerts, insufficient contextual relevance, and poor workflow interpretability identified as primary contributors [34]. Van De Sijpe et al. similarly reported 88.2% override rates for very severe DDI alerts in a hospital CDSS, attributing non-actionable alerts primarily to overly broad screening intervals and insufficient patient-specific contextualization [35]. The SafeRx prioritization framework incorporated mechanism-aware enrichment, never-event classification, and structured tier-based stratification to improve CDS interpretability within a high-density environment containing over 9900 consolidated interaction pairs. Prioritization logic was not generated through autonomous AI prediction, but through provenance-aware semantic enrichment combined with deterministic propagation logic and pharmacist-supervised moderation workflows.

At the same time, the conservative best-tier escalation policy entails an explicit operational trade-off that warrants reflection. The Tier 1 fraction is higher in the consolidated layer (46.86%) than at the RCP enrichment stage (14.50%). As detailed in Section 3.4, this difference is computed across different populations and primarily reflects the inclusion of inherently high-acuity specialized sources (notably OpenFDA regulatory boxed-warning and toxicity records) rather than cross-source best-tier escalation, which applies only to the small multi-source minority of pairs. As Tier 1 corresponds to interruptive hard-stop handling, this elevated Tier 1 fractionconcentrates a larger share of the canonical corpus within the most interruptive alerting band and therefore carries a latent risk of contributing to the alert fatigue that tier-based prioritization is specifically intended to mitigate. This trade-off reflects a deliberate sensitivity-favoring design choice, in which preserving genuine high-risk interaction signals during canonical deduplication was prioritized over minimizing the number of high-tier alerts, on the rationale that suppressing a true high-risk interaction constitutes a higher-consequence error than over-escalating a lower-risk one.

Several architectural features are intended to bound the resulting burden. The reported proportions characterize the composition of the knowledge base rather than the per-encounter alert rate, since at the point of care only the subset of canonical pairs matching a patient’s actually co-prescribed substances is surfaced. Only Tier 1 triggers hard-stop interruption, whereas Tier 2 and Tier 3 are handled through acknowledgment-based and passive non-interruptive mechanisms, respectively; and never-event flags together with mechanism-aware annotations enable further triage within the Tier 1 band. In addition, the pharmacist-supervised governance workflow permits longitudinal review and downgrading of over-escalated canonical pairs, supporting continuous recalibration of the prioritization layer. Nonetheless, the net effect of this escalation policy on interruptive alert burden and prescriber override behavior was not evaluated in the present study and should be assessed prospectively, ideally in conjunction with patient-specific contextualization, which has been identified as a principal determinant of alert actionability.

Mechanism-aware semantic enrichment represented an additional central component of the harmonized interaction infrastructure. The enriched corpus incorporated pharmacodynamic, pharmacokinetic, toxicologic, and physicochemical mechanism annotations including QT prolongation, CYP-mediated interactions, nephrotoxicity, hepatotoxicity, bleeding risk, respiratory depression, and intravenous incompatibility categories. Hu et al. introduced the MecDDI platform as a mechanism-oriented interaction resource operationalizing DDI knowledge into mechanistically interpretable layers including CYP450 modulation, transporter-mediated effects, and pharmacodynamic antagonism [36]. Our platform emphasized explainable interaction interpretation through semantically structured mechanism annotations and severity-aware prioritization rather than isolated binary interaction detection, consistent with the growing transition toward multimodal AI-assisted infrastructures integrating knowledge graphs, NLP pipelines, and context-aware CDS architectures [23,37].

The semantic enrichment framework implemented within SafeRx differs conceptually from fully predictive black-box AI systems. Rather than attempting autonomous de novo interaction prediction, the platform focused on provenance-aware semantic augmentation and operational structuring of existing pharmacologic evidence layers. This distinction is clinically relevant because many contemporary AI-driven interaction systems remain limited by insufficient explainability, poor auditability, or lack of transparent evidence attribution. By comparison, the SafeRx architecture preserved source-aware traceability, mechanism-linked annotations, and pharmacist-reviewable interaction representations throughout the enrichment workflow.

The predominance of GPT-assisted semantic representations within the provenance architecture supports the feasibility of integrating large language model–assisted semantic structuring into medication-safety infrastructures when combined with deterministic enrichment logic and expert-supervised governance mechanisms. This transparency-oriented architecture represents a fundamental architectural distinction from widely used commercial DDI knowledge bases, where interaction evidence origin, editorial curation criteria, and inclusion policies frequently remain undisclosed to end users. In contrast, SafeRx preserves explicit source attribution at the individual record level, enabling transparent differentiation between regulatory document-derived interaction content, AI-assisted semantic structuring, and propagated pharmacologic annotations throughout the entire knowledge graph. Rather than replacing authoritative pharmacologic knowledge, the platform operationalizes and harmonizes validated evidence from established regulatory and clinical sources within a semantically structured and clinically navigable framework.

To position SafeRx relative to established medication-safety resources, Table 5 summarizes a qualitative comparison with widely used drug-interaction and drug-information systems across the dimensions most relevant to the present contribution [38,39,40,41]. SafeRx is not intended to replace these resources, several of which provide extensive, professionally curated content. Rather, its distinguishing contribution lies in unifying heterogeneous medication-safety domains within a single provenance-aware canonical framework featuring record-level source attribution and open, pharmacist-supervised governance. Prior comparative evaluations have repeatedly documented substantial cross-platform variability in interaction classification and coverage, together with frequently undisclosed editorial criteria among commercial knowledge bases [6,9,27,32], which further motivates the transparency-oriented and interoperable design emphasized here.

The integration of specialized medication-safety domains constituted a distinguishing feature of the platform. Conventional interaction-checking systems frequently focus on pharmacokinetic or pharmacodynamic interaction detection while leaving reproductive safety resources, regulatory toxicity overlays, and physicochemical compatibility information distributed across separate repositories. SafeRx operationally incorporated FDA boxed warnings, LactMed-derived breastfeeding safety annotations, and Stabilis-derived intravenous compatibility data directly into the canonical substance-level workflow, extending the platform beyond conventional interaction-checking toward a unified multimodal medication-safety environment. Comparative lactation-safety analyses demonstrated that pharmacovigilance repositories such as FAERS and curated resources such as LactMed^®^ frequently differ in terminology structure, adverse-event categorization, and data standardization despite addressing the same substance-level safety concerns, emphasizing that spontaneous reporting systems function primarily as exploratory signal-detection environments while curated lactation resources provide more specific clinically interpretable guidance [22]. The intravenous compatibility layer may be particularly relevant given that physicochemical incompatibilities remain substantially underrepresented in conventional interaction infrastructures despite their importance in hospital-based intravenous administration workflows. The representative phenytoin workflow illustrated how concentration-specific compatibility data, precipitation observations, regulatory safety overlays, and lactation safety annotations can be simultaneously accessed within a single substance-centered clinical interface, demonstrating practical multimodal integration capabilities at the operational level.

The incorporation of pharmacist-supervised human-in-the-loop governance mechanisms constitutes an additional important aspect of the platform. The validation framework enabled structured review, approval, rejection, and modification of enriched interaction records prior to publication within the validated interaction layer, with all moderation actions preserved within an auditable append-only structure maintaining reviewer identity and before-and-after interaction snapshots. Eguia et al. emphasized that ontology-based normalization, semantic graphs, and hybrid human–AI workflows substantially improve clinical interpretability within medication-related decision systems [42]. The governance architecture combined automated extraction and enrichment with pharmacist-supervised clinical verification, representing an expert-augmented decision-support environment rather than a fully automated interaction engine.

From a translational perspective, the multimodal architecture may support pharmacist-oriented, pharmacovigilance, educational, and CDS-related applications across inpatient polypharmacy settings, intensive care environments, and high-risk intravenous administration workflows [17,43,44]. The operational distinction between high-priority interruptive alerts and lower-priority informational interactions may support more interpretable prioritization compared with non-stratified alert systems, while the open-access web interface enables transparent exploration and longitudinal community-based refinement of integrated medication-safety knowledge.

### Limitations and Future Directions

Several limitations should be acknowledged. The present study should be regarded as an exploratory, pre-implementation evaluation of the final SafeRx platform prior to prospective clinical validation. SafeRx has not undergone prospective validation in real-world clinical settings, and its impact on medication-error reduction, alert acceptance and override rates, clinical workflow efficiency, prescribing behavior, pharmacist interventions, and patient outcomes therefore remains to be determined. Accordingly, the integrated content, aggregated from authoritative regulatory, pharmacologic, and pharmacovigilance sources, should be regarded as comprehensive, provenance-referenced decision-support information of an indicative and advisory nature, intended to support rather than replace independent clinical and pharmacological judgment. The semantic enrichment architecture remains dependent on source quality and narrative consistency, and semantic variability may persist across heterogeneous interaction descriptions despite pharmacist-supervised moderation workflows. Patient-specific contextual variables such as renal function, laboratory values, and longitudinal medication histories were not incorporated, and the current implementation represents a harmonized knowledge environment rather than a fully integrated bedside prescribing architecture.

Furthermore, the current identity-resolution layer does not perform structure-aware unification of salt forms and stereoisomers onto a shared parent molecule; because matching relies on the full InChIKey and on lexical normalization rather than on cheminformatic parent resolution, interaction and safety knowledge attached to one salt or stereoisomer is not automatically propagated to chemically related forms, which may fragment substance-level aggregation.

In addition, output validity at the extraction stage is currently enforced post hoc rather than through a server-side JSON schema or a fixed decoding seed, controlled vocabularies are enforced in application logic rather than as database-level constraints, and no automated span-level verifier confirms that each extracted value is literally present in the source text. A quantitative extraction grounding rate was therefore not formally measured in the present exploratory study.

Although the conservative best-tier policy was designed to avoid suppressing high-risk interaction signals, the elevated Tier 1 fraction, driven mainly by the inclusion of high-acuity regulatory sources rather than by escalation across records, may overestimate actionable severity at the point of care and was not evaluated for its effect on interruptive alert burden or prescriber override rates. The high-acuity OpenFDA-derived regulatory layer represents the principal target for context-aware de-escalation, and the optimal balance between sensitivity and alert specificity remains to be established through prospective evaluation.

The tier validation was a blinded retrospective re-classification against the system-assigned tiers rather than a prospective evaluation against clinical outcomes, and was restricted to the GPT-derived RCP tiers. The single observed discordance suggests that boundary cases between adjacent severity tiers may represent a residual source of classification uncertainty. Continuous in-platform pharmacist review is at an early, pilot stage, and broader prospective validation across additional evidence sources and clinical settings remains necessary.

A minor limitation of the harmonization workflow was the inability to resolve one Stabilis compatibility pair (gelatin–vancomycin hydrochloride) because gelatin lacked a canonical substance mapping within the master registry.

Future work may explore FHIR-enabled EHR integration, extension of multilingual harmonization pipelines, graph-based pharmacovigilance querying, and prospective clinical validation studies quantifying the platform’s impact on medication-error reduction, alert acceptance and override rates, and clinical workflow efficiency. In addition, future iterations of the identity-resolution framework may address salt-form and stereoisomer fragmentation through InChIKey skeleton matching combined with ChEBI conjugate-acid/base and enantiomer relations and UNII parent/preferred-substance hierarchies, accompanied by a precision/recall evaluation on a curated salt and stereoisomer reference set.

Translating SafeRx into clinical practice will require addressing several practical dimensions beyond the harmonized knowledge layer presented here. Interoperability should follow established healthcare-data standards; exposing canonical substances and interaction tiers as HL7 FHIR resources, bound to standardized clinical terminologies, would enable integration into electronic health records through SMART on FHIR and CDS Hooks [45,46]. Clinical actionability further depends on patient-specific contextualization, incorporating renal and hepatic function, age, pregnancy status, and the active medication list, to suppress non-actionable alerts and mitigate alert fatigue [35]. Because such deployment would process identifiable health data, it must comply with the EU General Data Protection Regulation [47], applying data minimization, an explicit lawful basis, and pseudonymization. Finally, a clinical-grade SafeRx would likely constitute medical device software under the EU Medical Device Regulation [48], while its AI-assisted components would fall within the high-risk category of the EU Artificial Intelligence Act [49], requiring conformity assessment, data-quality governance, transparency, and human oversight. The present study therefore positions SafeRx as a pre-clinical knowledge infrastructure, with standards-based interoperability, patient-context integration, privacy-by-design, and regulatory conformity identified as the principal milestones toward clinical implementation.

## 5. Conclusions

SafeRx demonstrates the feasibility of constructing a provenance-aware, substance-centric pharmaceutical knowledge infrastructure capable of consolidating heterogeneous medication-safety resources, including drug–drug interaction data, regulatory toxicity overlays, LactMed-derived lactation safety information, and intravenous compatibility records, within a unified canonical framework. The platform integrated 2190 canonical substances, 9932 consolidated interaction pairs, and over 10,000 specialized safety records through ontology-aware normalization, AI-assisted semantic enrichment, and conflict-aware harmonization workflows, while maintaining explicit source attribution and enrichment provenance throughout.

The tier-based prioritization architecture, mechanism-aware annotations, never-event classifications, and pharmacist-supervised governance workflows collectively represent an expert-augmented approach to medication-safety decision support, distinct from fully automated or editorially opaque interaction systems. By operationally incorporating complementary safety domains frequently absent from conventional interaction checkers, SafeRx extends beyond traditional DDI infrastructure toward a multimodal clinical decision-support environment. Overall, canonical harmonization with provenance tracking, combined with explainable semantic enrichment and pharmacist-led governance, may offer a practical and scalable foundation for next-generation medication-safety infrastructures.

## Figures and Tables

**Table 1:** Canonical identifier harmonization overview.

Category	Count	%
Identifier type		
CAS	2290	24.20
UNII	2278	24.07
PubChem	1847	19.52
InChIKey	1824	19.28
ChEBI	1224	12.93
Mapping source		
FDA UNII	5015	53.0
Master-registry mappings	2796	29.5
ChEBI	1526	16.1
OpenFDA	108	1.14
LactMed	18	0.19

**Table 2:** Major mechanism categories.

Mechanism	Count	%
Intravenous incompatibility	1425	33.03
Precipitation	1131	26.22
Bleeding risk	840	19.47
Respiratory depression	378	8.76
QT prolongation	244	5.66

**Table 3:** Final consolidated operational tiers.

Tier	Count	%
Tier 1	4630	46.86
Tier 2	2118	21.44
Tier 3	3132	31.70

**Table 4:** Infrastructure-scale characteristics, update mechanisms, and database-level performance metrics of the SafeRx platform.

Characteristic	Value
Backend/application stack	Managed PostgreSQL 17 (Supabase); .NET 10, EF Core 10 (Npgsql)
Schema reproducibility	56 version-controlled migrations
Database footprint	353 MB
Schemas/tables/indexes	18/120/372
Total stored rows	≈1.04 million
Canonical substances/identifier mappings	2190/9463 (2016 mapped, 92.05%)
Consolidated interaction pairs	9932 (Tier 1: 4630; Tier 2: 2118; Tier 3: 3132; untiered: 52)
Specialized safety records	514 boxed warnings; 1895 lactation; 9088 IV compatibility
Materialized views	rcp.search_index (40,057 rows); master.interaction_summary (9932); atc.navigation_tree
Search indexing	Trigram GIN (idx_search_index_term_trgm) + B-tree prefix (idx_search_index_term_prefix)
Update cadence	Materialized views refreshed concurrently, daily 03:00 UTC (pg_cron); incremental sync gated by ANMC version + SHA-256 content hash; full re-acquisition on demand
Materialized-view refresh time (measured) ^1^	rcp.search_index: mean 2.4 s (1.6–4.3 s, *n* = 158); atc.navigation_tree: mean 0.6 s (0.3–0.8 s, *n* = 172); all recorded runs succeeded
Representative query latency (DB-level) ^2^	Ranked search 171.6 ms; substance detail 12.2 ms
Concurrency/batching controls	Concurrent LLM ≤ 3, PDF conversions ≤ 5, 150 ms inter-page crawl delay; batched DB writes

^1^ Measured from pg_cron run history (cron.job_run_details); database-side materialized-view refresh only, not full-pipeline or application-level performance; ^2^ Single-query PostgreSQL EXPLAIN ANALYZE execution times (warm cache); not application-level latency or throughput. ANMDMR, Romanian National Agency for Medicines and Medical Devices; DB, database; EF Core, Entity Framework Core; GIN, Generalized Inverted Index; IV, intravenous; LLM, Large Language Model; MB, megabytes; UTC, Coordinated Universal Time.

**Table 5:** Qualitative comparison of SafeRx with selected medication-safety resources, based on publicly documented platform capabilities.

Feature	SafeRx	DrugBank	Medscape	Lexidrug	Micromedex
DDI information	Yes	Yes	Yes	Yes	Yes
Regulatory warning information	Yes (OpenFDA boxed warnings)	Label-derived (FDA/Health Canada); no dedicated boxed-warning field documented	Drug-monograph warnings	Proprietary drug warnings	Proprietary drug warnings
Lactation-related information	Yes (LactMed)	Not a dedicated integrated source layer	Within drug monographs; not a dedicated database layer	Available in drug information resources	Available in drug information resources
Intravenous compatibility information	Yes (Stabilis)	Not identified	Not identified	Yes (Trissel’s IV Compatibility)	Yes (Trissel’s 2/IV INDEX)
Canonical cross-source substance harmonization	Yes	Structured drug data/API	Not publicly described	Proprietary/internal	Proprietary/internal
Explicit source/provenance layer	Yes	Not publicly documented	Not publicly documented	Not publicly documented	Not publicly documented
AI-assisted semantic enrichment provenance	Yes	Not publicly documented	Not publicly documented	Not publicly documented	Not publicly documented
Expert/pharmacist-supervised governance workflow	Yes (pharmacist-supervised, auditable)	Manual expert curation	Editorial curation	Proprietary editorial curation	Proprietary editorial curation
Open web access	Yes	Free academic web access; commercial API	Yes	Subscription-based	Subscription-based
Multimodal integration of DDI, regulatory warnings, lactation, and IV compatibility	Yes	Partial	Partial	Yes (proprietary)	Yes (proprietary)

Ratings reflect publicly documented platform capabilities as of 2026. For the multimodal-integration row, “Yes” indicates that all four listed safety domains (drug–drug interactions, regulatory warnings, lactation, and intravenous compatibility) are integrated within the platform; “Partial” indicates that some but not all of these domains are addressed, for DrugBank and Medscape, drug–drug interaction and regulatory-warning information are present, whereas intravenous compatibility is not identified and lactation is not provided as a dedicated source layer; and “Yes (proprietary)” indicates that all four domains are available but within closed, subscription-based systems. Entries marked “not publicly documented” or “not identified” denote features not described in public platform documentation rather than confirmed absence. Commercial systems provide extensive, professionally curated content. Cross-platform variability and editorial opacity among commercial knowledge bases have been documented previously. FDA, U.S. Food and Drug Administration; API, Application Programming Interface; IV, intravenous; DDI, drug–drug interaction.

## Data Availability

The raw data supporting the conclusions of this article will be made available by the first author on request.

## Supplementary Materials

The following supporting information can be downloaded at: https://www.mdpi.com/article/10.3390/bioengineering13070763/s1, Supplementary Material S1, Prompt for SmPC information extraction in JSON format; Supplementary Material S2, Pharmacist-reviewed canonical substance list (*n* = 200), including conformance to INN nomenclature and the 18 corrected name variants; Supplementary Material S3, Blinded tier-validation dataset (*n* = 90 interactions): independent reviewer tiers, system tiers, and confusion matrix.

## Abbreviations

The following abbreviations are used in this manuscript:

Abbreviation	Full term
AI	Artificial Intelligence
ANMDMR	National Agency for Medicines and Medical Devices of Romania
ATC	Anatomical Therapeutic Chemical
CAS	Chemical Abstracts Service
CDS	Clinical Decision Support
ChEBI	Chemical Entities of Biological Interest
CYP	Cytochrome P450
DCI	Denumire Comună Internațională
DDI	Drug–Drug Interaction
EHR	Electronic Health Record
FAERS	FDA Adverse Event Reporting System
FHIR	Fast Healthcare Interoperability Resources
FDA	Food and Drug Administration
GPT	Generative Pre-trained Transformer
ICU	Intensive Care Unit
INN	International Nonproprietary Name
IV	Intravenous
JSON	JavaScript Object Notation
LLM	Large Language Model
NLP	Natural Language Processing
OMOP	Observational Medical Outcomes Partnership
PCORnet	Patient-Centered Outcomes Research Network
PDF	Portable Document Format
RCP	Summary of Product Characteristics
SNOMED CT	Systematized Nomenclature of Medicine Clinical Terms
UNII	Unique Ingredient Identifier
