# Supplementary material for: SafeRx: A Canonical Pharmaceutical Knowledge Integration Framework for Multimodal Medication Safety
Source: Bioengineering (Basel). 2026 Jun 30;13(7):763. doi: 10.3390/bioengineering13070763 (PMC13405002; doi:10.3390/bioengineering13070763)
Supplement: Supplementary file 1 [file bioengineering-13-00763-s001.zip › Supplementary Material S1 bioengineering-4362006.pdf]

Prompt for SmPC Information Extraction in JSON Format

This supplementary material presents the structured prompt used for extracting clinically relevant information from Summary of Product Characteristics (SmPC) documents into a standardized JSON output.

Instructions

Please extract the information from the Summary of Product Characteristics (SmPC) corresponding to the active substance [{CommonInternationalName}], from the following sections:

- Section 4.5 – Interaction with other medicinal products and other forms of interaction
- Section 4.3 – Contraindications
- Section 4.6 – Fertility, pregnancy and lactation
- Section 4.8 – Undesirable effects

Return the data exclusively in valid JSON format, using the following structure:

```
{
  "substance": "Name of the analyzed substance",

  "interactions": [
    {
      "substance_or_class": "Name of the substance or pharmacological class",
      "interaction_type_effect": "Description of the interaction or effect",
      "recommendation": "Recommendation regarding use",
      "risk": "Minor / Medium / Major"
    }
  ],

  "other_information": {
    "contraindications": {
      "description": "Text extracted from section 4.3",
      "recommendation": "Clinical recommendation or note"
    },
    "pregnancy_first_trimester": {
      "description": "Text extracted from section 4.6",
      "recommendation": "Recommendation"
    },
    "pregnancy_second_third_trimester": {
      "description": "Text extracted from section 4.6",
      "recommendation": "Recommendation"
    },
    "lactation_newborns_premature_infants": {
      "description": "Text extracted from section 4.6",
      "recommendation": "Recommendation"
    },
    "very_common_adverse_reactions": {
      "description": "List of very common adverse reactions",
      "recommendation": "Recommendation or note"
    },
    "common_adverse_reactions": {
      "description": "List of common adverse reactions",
      "recommendation": "Recommendation or note"
    }
  }
}
```

Important notes

No.	Instruction
1	If no information is available in the SmPC for one of the sections, complete the "description" field with: "Not reported in the SmPC".
2	The "substance" field must contain the exact name of the main substance from the SmPC.
3	Preserve the original text as faithfully as possible in the "description" fields.
4	In the "recommendation" fields, formulate a clear, concise, and clinically useful summary.
5	Do not introduce information that is not present in the SmPC.
6	Do not add explanations outside the JSON structure.
7	Ensure that the final output is valid JSON.

The notation {CommonInternationalName} represents a programmatic variable used in our extraction pipeline. It is dynamically replaced with the specific International Nonproprietary Name (INN), such as *vancomycin* or *amoxicillin*, of the active substance being queried during execution.
